# Doubtful Justification of the Gluten-Free Diet in the Course of Hashimoto’s Disease

**DOI:** 10.3390/nu14091727

**Published:** 2022-04-21

**Authors:** Małgorzata Szczuko, Anhelli Syrenicz, Katarzyna Szymkowiak, Aleksandra Przybylska, Urszula Szczuko, Jakub Pobłocki, Danuta Kulpa

**Affiliations:** 1 Department of Human Nutrition and Metabolomics, Pomeranian Medical University, 71-460 Szczecin, Poland; 55664@student.pum.edu.pl (K.S.); urszula.szczuko@gmail.com (U.S.); 2 Department of Endocrinology, Metabolic Diseases and Internal Diseases, Pomeranian Medical University, 70-252 Szczecin, Poland; anhelli.syrenicz@pum.edu.pl (A.S.); jakub.poblocki@pum.edu.pl (J.P.); 3 Department of Internal Medicine and Diabetology, Poznan University of Medical Sciences, 60-834 Poznań, Poland; 81628@student.ump.edu.pl; 4 Department of Genetics, Plant Breeding and Biotechnology, Faculty of Environmental Management and Agriculture, West Pomeranian University of Technology, 71-434 Szczecin, Poland; danuta.kulpa@zut.edu.pl

**Keywords:** Hashimoto’s disease, gluten-free diet, gluten, anti-TPO, anti-TG

## Abstract

The popularization of the gluten-free diet brings with it a fashion for its use, which can harm the treatment of Hashimoto’s disease. The few studies in this regard do not confirm positive changes resulting from a gluten-free diet. At the same time, the presence of other comorbid autoimmune diseases in this group of patients is increasing. This may have important implications for the interpretation of test results and the need for a gluten-free diet in some patients. In this review, the PubMed database was searched for links between a gluten-free diet, Hashimoto’s disease, and autoimmune diseases. When analyzing the available literature, we found no basis for introducing a gluten-free diet for the standard management of Hashimoto patients. The recommended diet is instead an anti-inflammatory diet that levels the supply (to compensate for deficiencies) of vitamin D, iodine, and selenium, which are found in plant products rich in polyphenols, antioxidants, and omega-3 fatty acids, as illustrated in this article.

## 1. Introduction

Patients with Hashimoto’s disease (HD) may present diverse clinical manifestations. Attention is drawn to the high prevalence of undiagnosed celiac disease in patients with Hashimoto’s thyroiditis and the relationship between the two autoimmune disorders in these patients. Metagenomic and other studies in healthy and diseased individuals reveal that reduced biodiversity and changes in the composition of the gut microflora are associated with a variety of inflammatory conditions, including asthma, allergies, inflammatory bowel disease (IBD), type 1 diabetes and obesity [1]. The appropriate composition of microbiota is also a factor determining the proper function of the thyroid gland [2]. Microbes affect thyroid hormone levels by regulating iodine intake, degradation and enterohepatic circulation [3]. The development of autoimmune diseases of the thyroid gland is most often explained by the mechanisms of molecular mimicry, i.e., the appearance of autoreactive clones of T and B lymphocytes as a result of an immune cross-response to homologous bacterial or viral antigens [4]. Another explanation is that tissue transglutaminase serum immunoglobulin G (IgG) and gliadin immunoglobulin A (IgA) antibodies are significant predictors of anti-thyroid antibodies in patients with Hashimoto’s thyroiditis.

Increased levels of thyroid stimulating hormone (TSH) may positively correlate with vitamin D deficiency. In addition, seleno-methionine supplementation is considered among patients with autoimmune thyroid diseases, which inhibits the production of anti-thyroid peroxidase (TPO) antibodies [5,6,7]. Therefore, we will discuss supplementation in more detail later in the manuscript.

There is ample evidence of a strong association between HD and several immune mediated diseases. Some of these conditions share human leukocyte antigen (HLA) haplotypes. HLA haplotypes and non-HLA alleles, e.g., cytotoxic antigen 4 T lymphocytes (CTLA-4), may underlie pathogenesis [8]. The incidence of autoimmune thyroid disease is higher among patients with gluten/wheat hypersensitivity without celiac disease and those with dermatitis herpes. Moreover, the incidence of celiac disease (CD) is higher among patients over 65 years of age with autoimmune thyroid disease.

It can therefore be assumed that an autoimmune reaction occurs when several factors are present, including genetic predisposition, environmental factors that activate gene expression (infection, food allergies, chronic stress and an increase in cortisol, pregnancy and birth that changes the hormonal balance) and leaky gut syndrome, which interferes with the ability of the immune system to self-regulate. The hormonal response to stress by activating the hypothalamic-pituitary-adrenal axis causes the redirection of the immune response from type 1 T helper (Th1) to type 2 T helper (Th2), which suppresses cellular immunity and enhances the humoral condition, contributing to the development of HD [9].

So far, no review has been published describing the effect of a gluten-free diet on the course of Hashimoto’s disease. Although its popularity continues to increase in this group of patients, its routine use remains debatable. The aim of the study was to discuss the impact, benefits and risks of the gluten-free diet in the course of Hashimoto’s disease.

## 2. Materials and Methodology

In this review, the PubMed database was searched for the key terms Hashimoto and diet (*n* = 17), and thyroid diseases and diet (*n* = 6). Later in the research, we searched for articles linking Hashimoto’s with celiac disease (*n* = 11) and with gluten (*n* = 11). We also searched the terms autoimmune thyroiditis and gluten-free (*n* = 50).

Hashimoto’s disease and the gluten-free diet were the joint topics of five articles, three of which described studies on adults not diagnosed with celiac disease and for whom this had not been ruled out before gluten elimination.

## 3. Occurrence and Causes of Hashimoto’s Disease

It is estimated that 5% of the world’s population suffers from Hashimoto’s disease, also known as chronic lymphocytic thyroiditis [10]. According to endocrinologists in Poland, problems with thyroid dysfunction affect about 22% of the population [11]. The etiology of the disease involves genetic and environmental factors that cause the loss of immunological tolerance to the body’s own cells. As a result of this process, the human body produces pro-inflammatory mediators, including cytokines, which lead to the formation of lymphocytic infiltrates and activate the apoptosis of thyroid follicular cells and the process of organ fibrosis. In the case of Hashimoto’s disease, an important role is played by genetic factors, in particular polymorphisms in the major histocompatibility complex—HLA and in the CTLA-4 gene [10]. Along with environmental factors, the disease has also been linked with pharmacological treatment (e.g., with interferon), chronic stress and excessive stimulation of cortisol secretion, ionizing radiation, and the patient’s diet and lifestyle [10,12,13]. An important component of the diet is selenium, which, through selenoenzymes, is involved in maintaining the homeostasis of thyroid hormones. Numerous animal studies have confirmed the correlation between the appropriate concentration of selenium in the body and the appropriate function of the thyroid gland [14]. Iodine also plays an important role in synthesizing thyroid hormones, and its slight oversupply increases the risk of autoimmune thyroid disease.

Although the exact mechanism is not fully understood, studies suggest that excess iodine may induce apoptosis of thyroid cells [7]. Excessive iodine intake (median urinary iodine excretion (MUIE) > 300 μg/L) could well become a serious public health concern because of its capacity to substantially increase subclinical hypothyroidism and autoimmune thyroiditis (AIT) rates [15]. In Poland, however, there is rarely excessive consumption of this element due to the slight amount of fish in the diets of Poles. In that context, the prophylaxis of salt iodization was introduced in Poland, which some researchers have since associated with the increase in the occurrence of chronic autoimmune thyroiditis (cAITD) [16]. Further to this, the environmental factors contributing to the disease include exposure to chemicals such as polyaromatic hydrocarbons or polyhalogenated biphenyls, which are commonly used in industry. Although there is evidence of a link between exposure to chemicals and the incidence of autoimmune thyroid disease, the exact mechanisms by which they are connected have not yet been established [12].

## 4. Diagnosis of Hashimoto’s Disease

Nonspecific symptoms of the disease make its diagnosis difficult. A genetic defect or a decrease in the activity of T lymphocytes increases the secretion of pro-inflammatory cytokines and reduces the production of cytokines responsible for immune tolerance [10]. In addition, B lymphocytes produce tissue-specific antibodies that are directed against thyroid antigens: thyroid peroxidase antibodies and thyroglobulin antibodies (anti-TPO and anti-thyroglobulin (TG) [17]. Hashimoto’s disease is diagnosed based on the clinical picture, elevated serum levels of anti-TPO and/or anti-TG antibodies, abnormal serum levels of thyroid hormones, and a characteristic ultrasound image of the thyroid gland [17]. The thyroid gland in Hashimoto’s disease, on an ultrasound, is characterized by reduced heterogeneous echogenicity and fibrosis [18]. One of the symptoms of hypothyroidism with Hashimoto’s disease is bradycardia, i.e., a reduced heart rate caused by decreased contractility of the heart chambers and increased peripheral resistance [19]. In adults, swelling of the subcutaneous tissue due to the accumulation of mucopolysaccharides may be observed in connection with hypothyroidism [20]. Hypothyroidism may also be accompanied by vitamin B12 and iron deficiency, causing anemia, nervous system disorders that may lead to depression, and weakening of intestinal motility causing constipation. Hashimoto’s disease can impair ability to reproduce [20]. The quality of life of patients also deteriorates due to frequent changes in their mood, the feeling of chronic fatigue, problems with concentration, and changes in appearance due to hair loss or weight gain [21]. It is worth adding that often, before developing into the full symptoms of hypothyroidism, so-called subclinical hypothyroidism is observed in many patients. For a significant portion of these, early diagnosis and implementing appropriate treatment can inhibit the progression of subclinical hypothyroidism [22].

## 5. Diseases Accompanying Hashimoto’s Disease

Hashimoto’s disease may coexist with other chronic diseases, including autoimmune diseases. On average, every fifth person with CD also has chronic lymphocytic thyroiditis [23]. According to studies, more than 17% of patients with type 1 diabetes also have Hashimoto’s disease [24]. Patients with type 1 diabetes and elevated levels of anti-TPO and anti-TG antibodies are 18 times more likely to develop hypothyroidism than patients who have not demonstrated the presence of these antibodies [23]. Chronic lymphocytic thyroiditis is also diagnosed in patients with polycystic ovary syndrome (PCOS). Hashimoto’s disease is the most common thyroid disease coexisting with polycystic ovary syndrome, affecting up to 70% of patients with PCOS [25].

Autoimmune polyglandular syndromes (APS), which may also coexist with Hashimoto’s disease, are composed of disease entities characterized by dysfunction of several endocrine glands, caused by a loss of immunological tolerance. These syndromes involve antibodies present in the blood and lymphocytic infiltration of the affected organs [26]. There are three basic forms of APS:

- APS 1 occurs with a frequency of 1:100,000 births, is usually diagnosed in childhood, and for its diagnosis, it is necessary to find Addison’s disease coexisting with at least one of two pathologies: hypoparathyroidism and/or mucosal and skin candidiasis [26]. Genetic factors play an important role in the development of APS. The disease is inherited in an autosomal and recessive manner. Patients struggling with autoimmune polyglandular syndrome often experience fertility problems that result from premature ovarian failure in women or primary testicular failure in men. In addition, patients may develop other autoimmune diseases such as type 1 diabetes, autoimmune hepatitis, alopecia areata, or CD [23];
- APS 2 occurs at a frequency of approximately 1:1000 births. Most often, its symptoms appear in adolescence. It is characterized by the coexistence of Addison’s disease with at least one of two diseases: autoimmune thyroid disease and/or type 1 diabetes [26]. These patients develop other autoimmune diseases, including those associated with non-endocrine diseases, e.g., celiac disease, ulcerative colitis, or neuropathy [23];
- APS 3 is diagnosed in the fourth decade of life. It is characterized by the coexistence of an autoimmune disease of the thyroid gland with another autoimmune disease(s) such as type 1 diabetes, gastritis, vitiligo, or alopecia areata [23]. Addison’s disease is not one of the components of APS 3.

These three types of the syndrome require pharmacological treatment of particular disease entities [26]. Besides this, Hashimoto’s disease is also often associated with mental disorders. Clinical trials confirm the relationship between an increased concentration of anti-thyroid antibodies and the occurrence of depression in patients. Obsessive-compulsive disorders are also more common in patients struggling with lymphocytic thyroiditis. In the general population, the prevalence of obsessive-compulsive disorders is estimated at 1–3%, while this disorder affects over 15% of patients with Hashimoto’s [27].

## 6. Treatment

Pharmacotherapy in Hashimoto’s disease, if necessary, is the most important element of treatment, while a properly balanced diet may be important in supporting the therapy by providing the right amounts of nutrients to produce thyroid hormones [28]. Pharmacological treatment consists of the use of levothyroxine in an appropriate dose, adjusted to the body weight and the degree of damage to the thyroid parenchyma [29]. Since Hashimoto’s is the most common autoimmune disease today, many scientists are studying the validity of using different elimination diets in patients suffering from chronic lymphocytic thyroiditis. According to studies, 5% of adults and 8% of children suffering from Hashimoto’s are also diagnosed with CD [30]. This is a much higher percentage than in the general population, where about 1% of the population struggle with CD [31]. The legitimacy of the gluten-free diet in Hashimoto’s disease has been investigated by many researchers. It has not been clearly demonstrated that gluten can stimulate the immune system to produce autoantibodies, which due to their structure, destroy thyroid tissue [32]. In this context, we set out to determine whether eliminating gluten from the diet of Hashimoto’s patients (regardless of the presence or absence of celiac disease) helps to alleviate the symptoms of the disease.

## 7. Gluten-Free Diet

The main principle of a gluten-free diet is to eliminate grains from the diet that are a source of gluten, i.e., all types of wheat, barley, rye, and oats (oats are often contaminated with other grains). The diet involves eliminating not only food that may contain gluten but also beverages, and even drugs or dietary supplements containing wheat, barley, or rye [33]. A gluten-free diet consists mainly of naturally gluten-free products, i.e., fruit, vegetables, meat, fish, legumes, nuts, dairy products, and eggs [34]. Naturally gluten-free cereals include corn, rice, millet, sorghum, and eragrostis tef [34]. Yet, the diet can be restrictive as gluten is often used as a filler in the food industry, e.g., in cold cuts, or as a food additive, e.g., malt [35]. It increases the flexibility and viscosity of cakes and bread [36]. Higher consumption of cereal products in the daily diet, including gluten, increases the risk of non-celiac gluten sensitivity (NCGS) and CD in the population, which is why Europeans are the most vulnerable [37]. The amount of gluten in the European diet is on average 10–20 g per day [38]. NCGS is a clinical entity characterized by the absence of celiac disease and wheat allergy in patients that trigger reproducible symptomatic responses to gluten-containing foods consumption [31]. For NCGS diagnosis, placebo-controlled gluten challenges must be carried out. Therefore, the exclusion of CD and wheat allergy (WA) for the diagnosis work-up of NCGS remains a key step due to the lack of biomarkers for NCGS diagnosis [37]. In addition to cases of CD, a gluten-free diet is also advised for people with gluten intolerance, wheat allergy, or Dühring’s disease [33]. Dermatitis herpetiformis (DH), also known as Duhring-Brocq dermatitis, is a chronic, recurrent disease, secondary to gluten hypersensitivity. DH patients rarely have gastrointestinal symptoms, but they generally present some degree of intestinal villous atrophy [38]. It affects predominantly Caucasians, more prevalent in Scandinavian countries and in the UK [39]. Dietary triggers such as gluten and highly fermentable oligo-, di- and mono-saccharides and polyols (FODMAP)-containing foods have been associated with worsening irritable bowel syndrome (IBS) symptoms. An extensive meta-analysis concluded that there is insufficient evidence to recommend a gluten-free diet (GFD) to reduce IBS symptoms. However, there is very low-quality evidence that a low FODMAP diet is effective in reducing symptoms in IBS patients [40].

Implementing a gluten-free diet, just like any other elimination diet, is associated with a high risk of dietary deficiency. Gluten-free products, compared to their traditional counterparts, have much lower nutritional value [41]. The most common deficiencies in patients on a gluten-free diet are vitamins B or D, calcium, and iron. Insufficient magnesium, zinc, selenium, and copper intake is also often observed [33]. Since gluten-free food production is a complicated process, gluten-free products are often highly processed and contain more fats and carbohydrates than their traditional counterparts. Furthermore, patients who stop feeling discomfort after eating when switching to a gluten-free diet are more likely to eat meals, in general, which due to the high calorie content of some gluten-free products, may lead to obesity [40]. Buying gluten-free product substitutes has been shown to increase the cost of the daily diet by up to 30% compared to the conventional diet, and this cost may be even greater due to the need to select products clearly labeled gluten-free [41].

## 8. Structure and Absorption of Gluten

Gluten is a protein complex consisting mainly of gliadin and glutenin. Due to their large amounts of proline and glutamic acid, these proteins are classified as prolamines [42,43]. Protein hydrolysis in the human body is facilitated by the enzymes produced by the stomach and pancreas. The final products of protein digestion are free amino acids, dipeptides, and tripeptides, which are absorbed in the small intestine via active transport, i.e., using energy in the form of adenosine triphosphate [43]. Gluten peptides are relatively resistant to the process of proteolysis that takes place in the small intestine. As a result, gluten remains partially undigested. High-molecular-weight molecules remain in the intestinal lumen and may become a substrate for bacterial metabolism [44]. Polypeptides formed during the breakdown of gluten, as tissue transglutaminase undergoes deamination, result in the formation of deaminated gliadin peptides. Afterward, the gliadin is transformed into more immunogenic lysine through the process of transamidation. This process triggers an immune reaction in the body. In response, the body produces antibodies against tissue transglutaminase and deamidated gliadin peptides [45]. The newly formed peptides form complexes with the molecules of the HLA compatibility system, and they are then presented to the T lymphocytes. This results in the production of pro-inflammatory cytokines, which damage the intestinal mucosa [46]. This process causes flattening of the intestinal mucosa in patients suffering from celiac disease, with the disappearance of intestinal villi. Gliadin shows the highest toxicity amongst all gluten fractions. It is clinically important for the body’s immune response. There are different types of gliadin, which differ in their immunogenicity [47]. The most immunotoxic fragment of the gliadin protein is 33-mer. This fragment, among others, remains active in the intestine after gluten consumption [48].

## 9. Debatable Validity of Introducing the Gluten-Free Diet in Hashimoto’s Disease

The frequent coexistence of chronic lymphocytic thyroiditis and celiac disease led scientists to consider a gluten-free diet appropriate for patients with Hashimoto’s disease. The relationship between the occurrence of celiac disease and Hashimoto’s disease was attributed to the same genetic factor being responsible for both diseases [49]. Moreover, it was noted that both diseases are autoimmune. Several studies have since been carried out to test the effectiveness and safety of the gluten-free diet in patients with Hashimoto’s disease.

In 2018, Krysiak et al. [50] conducted a study to check the impact of a gluten-free diet on the process of autoimmunization and the function of the thyroid gland in patients with Hashimoto’s disease. The study lasted six months, with 34 euthyroid women (aged 20–45) participating. The participants were divided into two groups: those following a gluten-free diet and the others who did not eliminate gluten. The patients attended a follow-up visit every two months. The blood concentrations of thyrotropin, free triiodothyronine, anti-TPO, and anti-TG antibodies were measured in each participant. The concentration of antibodies against tissue transglutaminase (anti-tTG) was also tested, and the level of 25-hydroxyvitamin D was measured using immunological tests. It was found that the anti-TPO and anti-TG antibody levels decreased in patients following a gluten-free diet. However, because no small intestinal biopsy was performed, it is possible that patients might have had subclinical (non-symptomatic) coeliac disease.

Similar results were obtained in 2000 by Ventura [51]. Patients suffering from celiac disease and Hashimoto’s disease participated in his study. A significant decrease in anti-thyroid peroxidase antibody levels was observed among the participants. Yet, in the study conducted by Krysiak [50], the gluten-free diet did not affect the concentration of thyrotropin or any other thyroid hormone among the patients, causing just a slight reduction of thyroid autoimmunity. In the majority of patients, a decrease in the concentration of antibodies against tissue transglutaminase, as well as an increase in the level of 25-hydroxyvitamin D, was documented. Scientists cannot explain the mechanism responsible for the beneficial effect of a gluten-free diet on thyroid autoimmunity. All participants initially had low serum vitamin D levels, and in patients following a gluten-free diet, the serum vitamin D levels increased. The increase in vitamin D concentration likely lowered the levels of anti-thyroid antibodies in patients. Another reason for the decrease of anti-thyroid antibodies in patients may be that the gluten-free diet adequately supplied selenium. A gluten-free diet can have anti-inflammatory effects in itself, as adhering to a gluten-free diet decreases the anti-inflammatory cytokines, unlike a conventional diet, which modifies the cytokines into an inflammatory profile [52]. The results of these studies showed, however, that eliminating gluten did not affect the metabolism of thyroid hormones and thus had no direct effect on the functioning of the thyroid gland.

When discussing the impact of a gluten-free diet on Hashimoto’s disease, it is worth mentioning the research conducted by Riseh et al. [49] on the relationship between the thyroid hormones, anti-TPO, anti-TG antibodies, anti-tissue transglutaminase, and the levels of anti-gliadin antibodies. These studies indirectly support the use of a gluten-free diet in patients suffering from chronic lymphocytic thyroiditis.

A group of 82 women (20–50 years old) participated in the Riseh et al. study [49]. Some of the participants had Hashimoto’s disease (40 people) and the remaining were healthy. Patients with celiac disease, diabetes, cardiovascular diseases, those taking medications that may have affected test results, and those following a gluten-free diet were excluded. Each participant had their blood tested for concentrations of thyrotropin, thyroxine, and triiodothyronine. In patients suffering from Hashimoto’s, higher levels of anti-tissue transglutaminase and anti-gliadin IgA antibodies were observed compared to healthy patients. Yet, a higher concentration of anti-gliadin IgG antibodies was also observed in the control group. This analysis showed that the anti-thyroid antibody levels fluctuate depending on the anti-tissue transglutaminase and anti-gliadin antibodies. These studies confirmed the increased risk of celiac disease in patients with Hashimoto’s disease and the frequent occurrence of its asymptomatic form in this group of patients. It is worth mentioning that celiac disease may reduce iodine absorption, which may hinder the treatment of Hashimoto’s disease. We can also assume that implementing a gluten-free diet reduces the concentrations of anti-tissue transglutaminase and anti-gliadin antibodies, which in turn, could reduce the concentrations of anti-TPO and anti-TG. Reducing the concentration of anti-thyroid antibodies could limit the process of autoimmunization, thereby favorably influencing the course of Hashimoto’s disease.

Research on the effectiveness of the gluten-free diet in Hashimoto’s disease was also carried out by Kus et al. in 2016 [53] on 156 patients. Unlike the two studies cited earlier, this was conducted using a survey. The participants were of all ages and backgrounds. Almost 75% of the respondents declared they followed a gluten-free diet. Most (88%) received pharmacological treatment with levothyroxine. The survey results were collected and analyzed. The majority of participants who did not receive pharmacological treatment reported a decrease in blood thyroid-stimulating hormone (TSH) levels. A significant proportion of the respondents also declared a reduction in their symptoms of Hashimoto’s disease. The majority of the participants stated they did not experience digestive issues while following the diet, though 43.5% of respondents had such symptoms beforehand. According to this study, following a gluten-free diet had a beneficial effect on the course of Hashimoto’s disease. However, it is worth recalling this was only a questionnaire survey, and the responses were not verified by the researchers. The results of thyrotropin concentration blood tests were only self-declared values, which means the patients provided their values and the measurements were performed by various laboratories. Testing at different institutions makes it impossible to compare the results accurately and renders any such findings unreliable. Additionally, the patients were not educated on the principles of a gluten-free diet; their knowledge and actual elimination of gluten from the diet were not verified. The patients were also not tested for celiac disease.

Further to this, research on the validity of using elimination diets in patients with Hashimoto’s disease was also carried out by Konieczny et al. [54]. The study involved 209 adults, including 81 people with Hashimoto’s disease and 118 with celiac disease. All participants followed an elimination diet before their involvement in the study. The study retrospectively assessed the quality of life and health of the patients before and after implementing the elimination diet. For this purpose, two types of questionnaires were used. Patients suffering from Hashimoto’s completed the ThyPROpl questionnaire (thyroid-specific patient-reported outcome questionnaire in the Polish language), while patients suffering from celiac disease completed the CSI (Celiac Symptom Index) questionnaire. Based on the answers given by the study participants, implementing an elimination diet was found to reduce the severity of disease symptoms. In the participants following a gluten-free diet, the most frequent improvement was a decrease in the occurrence of digestive issues. The participants also declared a reduction in fatigue, less frequent mood changes, and improved concentration. Yet, it is worth noting that the group of participants suffering from Hashimoto’s disease was not diagnosed with celiac disease, but it cannot be ruled out that some of the respondents also suffered from undiagnosed celiac disease, which may have increased the impact that implementing a gluten-free diet had on improving their health [54]. The diet should be rich in antioxidants, i.e., vitamins A, C, E, polyphenols, and omega-3 fatty acids. At the same time, supplementing the diet with minerals such as selenium, io-dine, magnesium, zinc, and copper is more important for Hashimoto’s patients than eliminating gluten itself [55]. A statistically significant reduction in TSH after 12 months was found in a recent clinical study where 62 patients with Hashimoto’s disease were considered (including 32 on a gluten-free diet), but no such relationship was observed after 3 and 6 months of dieting. The patients were treated with L-thyroxine during this period, and that supplementation was considered the main cause of the reduction in TSH level. No statistically significant changes in the levels of thyroid hormones or antibodies were observed [56]. The authors ruled out the influence of the gluten-free diet on thyroid parameters in people with Hashimoto’s without celiac disease [56]. Although, thyroid-associated antibodies may respond to implementing a gluten-free diet in patients with coexisting celiac disease and autoimmune thyroid disease [57]. All analyzed studies are presented in the table below (Table 1).

A gluten-free diet reduces the concentration of anti-tissue transglutaminase antibodies, which correlates with the concentration of anti-thyroid antibodies, meaning that the reduction may contribute to reducing thyroid autoimmunization. According to the patients, eliminating gluten from their diet reduced their symptoms, especially those related to the digestive system. However, it did not change their concentrations of thyroid hormones, which are the direct causes of the symptoms of the disease. Here, we must note that among the participants of the studies conducted so far, celiac disease has not been ruled out, i.e., the participants have not undergone the diagnostic process for this disease.

## 10. Discussion

**Figure 1:**
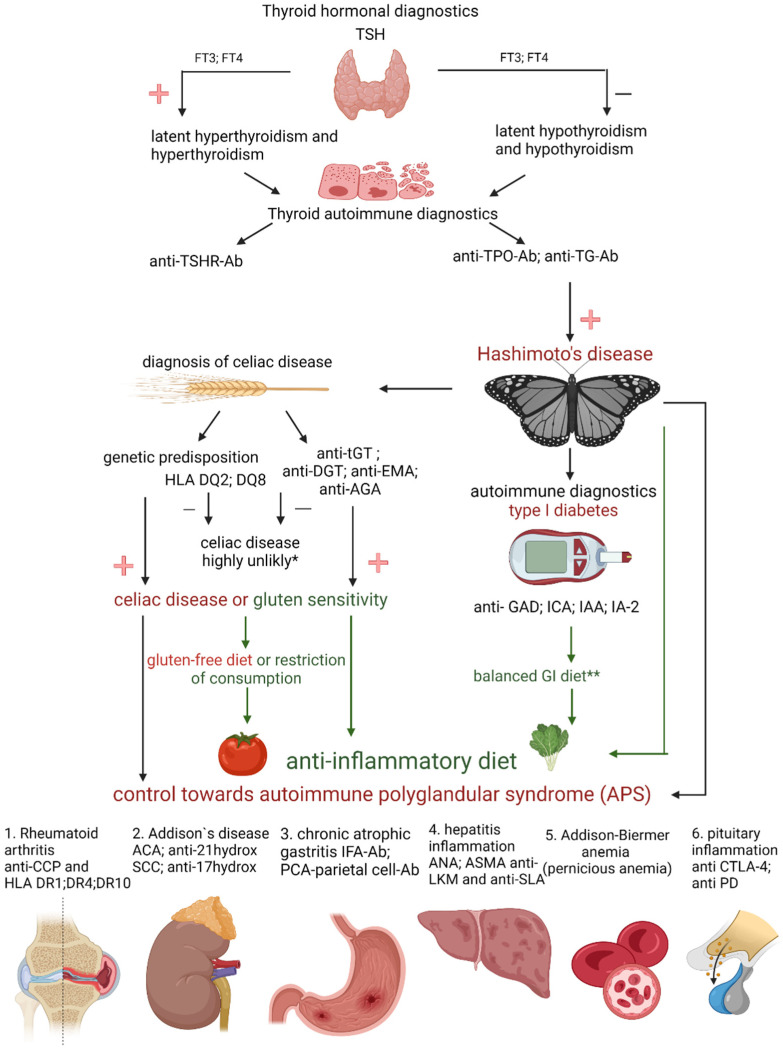
Hashimoto’s warrior alphabet. TSH—thyroid-stimulating hormone; FT3—free triiodothyronine; FT4—free tetraiodothyronine; anti-TSHR-Ab—thyrotropin antibodies; anti-TPO-Ab—antibodies to thyroid peroxidase; anti-TG-Ab—thyroglobulin antibodies; HLA DQ2, DQ8—human leukocyte antigen class II subregion: DQ2, DQ8; anti-tGT—anti-transglutaminase antibodies; anti-DGT—anti deamidated gliadin peptide antibodies; anti-AGA—anti-gliadin antibodies; anti-EMA—endomysial antibodies; anti-GAD—antibodies glutamic acid decarboxylase; ICA—pancreatic islet antibodies; IAA—insulin antibodies; IA-2—antibodies to tyrosine phosphatase; GI—glycemic index; anti-CCP—anti-cyclic citrullinated peptides; HLA DR1, DR4, DR10—human leukocyte antigen class II subregion: DR1, DR4, DR10; ACA—anticardiolipin antibody; anti-21hydrox—21-hydroxylase antibodies; SCC—squamous cell carcinoma; anti-17hydrox—17 hydroxylase antibodies; IFA-Ab —intrinsic factor antibodies; PCA—parietal cell antibodies (Ab); ANA—antinuclear antibodies; ASMA—antismooth-muscle antibody; anti-LKM—liver and kidney microsomal antigens; anti-SLA—anti-soluble liver antigen; anti CTLA-4—antibodies for cytotoxic T cell antigen 4; anti PD—programmed death monoclonal antibodies; −, minus; +, plus; * the biopsy is conclusive; ** the insulin dose is difficult to determine (created with BioRender.com, https://app.biorender.com/, accessed on 30 January 2022).

Based on the abovementioned research results, we can see there are potential benefits to a gluten-free diet in patients suffering from chronic lymphocytic thyroiditis. In some patients, the concentration of anti-thyroid antibodies decreased, and gastrointestinal symptoms were partially relieved. The presented studies confirm a significant correlation between the anti-thyroid antibodies, anti-gliadin antibodies, and glutamine transaminase. However, it is worth remembering that following a gluten-free diet, despite its potential benefits, has some risks. These are especially significant when the patient introduces an elimination diet on their own. There is currently no evidence that a gluten-free diet is beneficial in Hashimoto’s disease. It seems that gluten should only be eliminated by patients suffering from celiac disease or gluten sensitivity, which may coexist with Hashimoto’s disease [58]. As confirmed by various studies, such patients constitute 5–19% of HD patients. It is worth recalling that a gluten-free diet does not affect the concentration of thyroid hormones and thus does not tackle the biggest problem of patients with Hashimoto’s disease, i.e., the insufficient production of thyroid hormones. In Hashimoto’s disease, due to the ongoing inflammatory process in the body, an anti-inflammatory diet (mostly plant-based) should be implemented. To date, no studies have focused on the effects of a gluten-free diet in non-celiac patients with autoimmune thyroid disease. The incidence of celiac disease is higher among patients over 65 years of age with autoimmune thyroid disease [59], and it is higher among children with autoimmune thyroid disease (6.2%, confidence interval (CI) = 4.0–8.4%) compared to adults (2.7%, CI = 2.1–3.4%) [60]. The incidence of autoimmune thyroid disease is also high among patients with gluten/wheat hypersensitivity without CD [61,62] and those with dermatitis herpes [63]. Accordingly, the next stage of treatment in patients with Hashimoto’s should include screening for thyroglobulin antibodies (anti-TG), anti-deamidated gliadin peptid (anti-DPG), endomysial antibodies (EMA), and anti-gliadin antibodies (AGA) to exclude subclinical celiac disease and NCGS. AGA is also known to be less specific than EMA in serological evidence of CD. A diagram illustrating the recommended strategy is presented in Figure 1.

## 11. Conclusions

In summary, there is currently no evidence that a gluten-free diet is beneficial in Hashimoto’s disease. It seems that gluten should only be eliminated by patients suffering from celiac disease or gluten sensitivity, which may coexist with Hashimoto’s disease. A gluten-free diet does not affect the concentration of thyroid hormones and due to the ongoing inflammatory process in the body, an anti-inflammatory diet (mostly plant-based) should be implemented. Supplementing (to compensate for deficiencies) the diet with minerals such as selenium, iodine, magnesium, zinc, and copper is more important for Hashimoto’s patients than eliminating gluten itself. Patients with Hashimoto’s disease should be screened for other clinically relevant endocrine autoimmune diseases. After other autoimmune diseases have been ruled out, they should have regular follow-ups, as patients may still develop other autoimmune disorders over time.

In conclusion, the authors emphasize the importance of screening patients with Hashimoto’s disease for the presence of any secondary endocrine abnormalities. Studies conducted so far do not support the claim that HD patients absolutely should eliminate gluten from diet.

## Figures and Tables

**Table 1:** The studies.

Author, Year	Number of Patients (*n*)	Duration of the GFD Diet	Age and Gender W = Women M = Men	Exclusion Criteria	Inclusion Criteria	Effect	Comment
Riseh 2017 [49]	*n* = 82 HD group *n* = 40 CG group *n* = 42	GFD was notapplied	20–50 years W	GFD before the intervention celiac disease, diabetes, cardiovascular diseases	–	in HD higher levels of anti-tissue transglutaminase and anti-gliadin IgA antibodies were observed	these studies confirmed the increased risk of CD in patients with Hashimoto’s disease and the frequent occurrence of its asymptomatic form in this group of patients
Krysiak 2019 [50]	*n* = 34 GFD group *n* = 16 CG group *n* = 18	6 months	20–45 years W	GFD before the intervention + TRAb CD diabetes other chronic diseases	Euthyroid (0.4–4.5 mU/L) + anti-TPO (>100 U/mL) the reduced echogenicity	no change TSH and fT3 and fT4 ↧ anty-TPO and anty-TG	because no small intestinal biopsy was performed, it is possible that patients might have had subclinical (asymptomatic) coeliac disease
Ventura 2020 [51]	*n* = 180 CD *n* = 90 W including 11 person with HDCG group *n* = 90	6, 12, 24 months	age at diagnosis 10.1 years CD W = 61; M = 29 CG W = 60; M = 30 mean age 20.5 years	–	biopsy-confirmed CD	↧ anty-TPO no abnormality was found in serum levels of thyroid hormones or thyroid-stimulating hormone	GFD started early may prevent the other autoimmune diseases frequently associated with CD
Kus 2016 [53]	*n* = 156 all with HD	survey research	18–60 years W = 139 (89%); M = 17 (11%)	–	75% respondents with HD were treated with levothyroxine	the respondents stated no symptoms of the digestive system after GFD	taking measurements in different places by the patient makes it impossible to compare the results accurately and is unreliable
Konieczny 2019 [54]	*n* = 209 HD group *n* = 81 CD *n* = 118	survey research	18–60 years HD W = 81, CD W = 106; M = 12	–	–	respondents found a reduction in the incidence of digestive problems	biochemical studies have not been performed
Pobłocki 2021 [56]	*n* = 92	3, 6, 12 months	18–55 years W	GFD malabsorption syndromes, bariatric surgery diabetes, hypertension, coronary artery disease, active inflammation, use of gluco-corticosteroids	diagnosed cAITD ultrasound image anty-TPO anty-TG	level of TSH, fT3, fT4, anti-TPO and anti-TG-no differences were found after 3 and 6 months ↥ fT4 and ↧ TSH after 12 months	the authors ruled out the influence of the gluten-free diet on thyroid parameters in people with Hashimoto’s without CD

GFD, gluten-free diet; HD, Hashimoto’s disease; CG, control group; IgA, Immunoglobulin A; CD, celiac disease; TSH, thyroid-stimulating hormone; TRAb, Autoantibodies against TSH receptor; TPO, thyroid peroxidase; fT3, free triiodothyronine; fT4, free tetraiodothyronine; TG, thyroglobulin; cAITD, chronic autoimmune thyroiditis; ↧, decrease; ↥, increase.

## Data Availability

Not applicable.
